# Nutritional, Pharmacological and Industrial Applications of Mangosteen and Passion Fruit: A Review

**DOI:** 10.1002/fsn3.70574

**Published:** 2025-07-07

**Authors:** Muhammad Tayyab Arshad, Nosiba S. Basher, Nasir A. Ibrahim, Ali Ikram, Sammra Maqsood, Amara Rasheed, Feroza Naveed, Muhammad Waqar, Awais Raza, Sana Noreen, M. K. M. Al, Muhammad Ahmad, Mahreen Faqeer Hussain, Ali Jebreen, Ammar AL‐Farga

**Affiliations:** ^1^ Functional Food and Nutrition Program, Faculty of Agro‐Industry Prince of Songkla University Hatyai, Songkhla Thailand; ^2^ University Institute of Food Science and Technology The University of Lahore Lahore Pakistan; ^3^ Department of Biology, College of Sciences Imam Mohammad Ibn Saud Islamic University (IMSIU) Riyadh Saudi Arabia; ^4^ National Institute of Food Science and Technology University of Agriculture Faisalabad Faisalabad Pakistan; ^5^ Department of Food Science Government College University Faisalabad Faisalabad Pakistan; ^6^ Food Technology and Innovation Research Center of Excellence, School of Agricultural Technology and Food Industry Walailak University Nakhon Si Thammarat Thailand; ^7^ University Institute of Diet and Nutritional Sciences, the University of Lahore Lahore Pakistan; ^8^ Department of Physics, College of Sciences Imam Mohammad Ibn Saud Islamic University (IMSIU) Riyadh Saudi Arabia; ^9^ Department of Therapeutic Medical Nutrition, Faculty of Allied Medical Sciences Palestine Ahliya University Bethlehem Palestine; ^10^ Biochemistry in Department of Biological Sciences, College of Science University of Jeddah Jeddah Saudi Arabia

**Keywords:** anthocyanins, antioxidants, bioactive composites, flavonoids

## Abstract

The stunning nutritional and medicinal attributes of tropical fruits have propelled them to international fame. Passion fruit ( *Passiflora edulis* ) and mangosteen ( *Garcinia mangostana* ) are two examples of such fruits. This review discusses in depth their phytochemical composition, health benefits, and utility in the industrial sector. Mangosteen is an important dietary food and nutraceutical agent that exhibits high antioxidant, anti‐inflammatory, antibacterial, and anticancer properties because of its richness in xanthones, anthocyanins, and polyphenols. Passion fruit has the potential to serve as an antidiabetic, antihypertensive, and anticancer agent because it is rich in vitamins (A, B2, and C), carotenoids, and polyphenols. Owing to its antioxidant nature, mangosteen has applications in the cosmetics industry. Passion fruit, however, is extensively used in beverages, pastries, and sweets owing to its flavor and bioactive benefits. This review highlights the numerous uses of these fruits and demonstrates how they can contribute to the areas of sustainable agriculture, medicine, and nutrition. Perishability, limited cultivation, and underutilized by‐products (e.g., peels and seeds) are challenges that persist despite their benefits. Enhancing postharvest technologies, boosting agronomic practices, and valoring waste would make them more commercially viable. Enhancing extraction procedures, shelf life, and finding new applications in pharmaceuticals and functional foods are all directions in which future investigations need to focus to provide maximum social, economic, and health gains.

## Graphical abstract

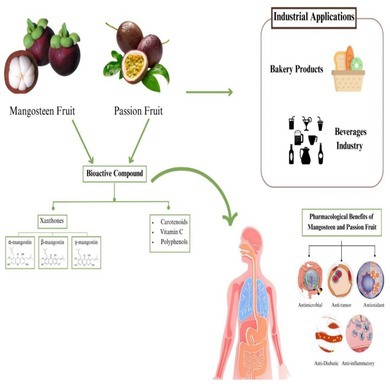

## 1 Introduction

Tropical fruit accounts for 62% of the world's supply of fresh fruit, as it fills a particular position in the global agricultural sector (Afzaal et al. 2022). Tropical fruits are grown in regions with hot and humid climates, including Asia, Europe, Latin America, Central America, the Caribbean, and Oceania. Asia produced 86% of the global output of tropical fruits between 2015 and 2017, making it the largest producer. By 2018, 235 million tons were produced, compared to 73.2 metric tons in 2010. Over the past decade, output has rapidly expanded in both domestic and foreign markets. Owing to Southeast Asia's unmatched diversity of genetic resources, many cultivated tropical fruit trees have been identified as having their origins there (Zimmerer 2023).

Owing to their significance in a balanced diet and their value as a source of revenue and nourishment for agricultural regions, few tropical fruits are being produced globally and traded more (Midin and Goh 2022). Household surveys show that up to 75% of income comes from these fruits. However, advancements in agricultural technology have significantly changed production trends (Palakawong and Delaquis 2018). Passion fruit and mangosteen are rapidly becoming well‐known tropical fruits (Ibrahim et al. 2016). Mangosteen is a translucent white fruit with a purple outer shell. It is naturally divided into larger segments that have inedible seeds when opened. Both fruits have distinct flavors and are delicious and nutritious. An adherent of the Clusiaceae genealogy, mangosteen is identified by its scientific name, *Garcinia mangostana* (*Ramirez* et al. 2017).

The mangosteen fruit, or *G. mangostana* L., ordinarily recognized as mangosteen, is one of the most popular fruits globally. A tropical fruit known as mangosteen is thought to have grown on Sunda Island and Moluccas (Palakawong and Delaquis 2018). Less frequent sources are Sri Lanka and Australia, but most of its production occurs in Asia, specifically in India, China, Bangkok, Malaysia, Hong Kong, Indonesia, Taiwan, Cambodia, and the Philippines. The Mediterranean, Central and South America, the US (particularly Puerto Rico), and a few others also experience output owing to perfect tropical or subtropical conditions (Carias 2023). Mangosteen is an apomictic plant that produces offspring without the use of fertilizer. Given that seeds grow only from the mother tissue, logically, all trees should be clonal (Midin and Goh 2022). Anthocyanins, xanthones, polyphenolics, and tannic acids are some of the bioactive constituents of mangosteen fruit extract, which is a favored nutritional supplement. These compounds can be used as functional food additives or medicinal agents, providing anti‐inflammatory, antimicrobial, and oxidative stress‐reducing effects (Tripathi et al. 2018). Mangosteen also contains terpenes, flavonoids, polyphenols, tannic acid, and specific vitamins (Eiselt et al. 2025).

According to Husen, Kalqutny, et al. (2017), Husen, Khaleyla, et al. (2017), and Husen, Winarni, et al. (2017) 80.9 g of water, 0.5 g of fat, 18.4 g of carbohydrates, 1.7 g of dietary fiber, 9 g of calcium, 14 mg of phosphorus, 0.4 mg of copper, 2 mg of vitamin C, 0.09 mg of thiamin, 0.06 mg of riboflavin, and 0.1 mg of niacin constitute 100 g of mangosteen fruit. The primary compounds in mangosteen pericarps are xanthones, which include tovophyllin A and B, α‐mangostin, γ‐mangostin, 8‐deoxygartanin, mangostanol, garcinone E, β‐mangostin, and mangostenones C, D, and E, as reported by Ansori et al. (2020). Mangosteen is the major xanthone derivative and exhibits numerous pharmacological properties such as antioxidant, anti‐inflammatory, and antidiabetic activities. Passion fruit, which belongs to the family Passifloraceae, is an economically important fruit crop cultivated worldwide (Tripathi et al. 2018). Passion fruit ( *Passiflora edulis* ) is a tropical fruit with a hard outer shell, golden pulp, and a seed‐filled interior. It has a sour flavor and is crisp and delicious. In the tropical and subtropical regions of South America, most Passiflora species are indigenous (Fischer and Miranda 2021).

Brazil is the center of diversity in Passifloraceae, also known as granadillas, purple granadillas, yellow passion fruits, maypop flowers, apricot vines, wild passion flowers, and Jamaican honeysuckles (Deshmukh et al. 2017). Of the 400 known species, 50–60 bear edible fruits, with most unknown outside their origin. *Passiflora* produces unisexual flowers in the leaf axils (*Stafne* 2022). The fruit has an oval or spherical shape, featuring a firm, sleek, wax‐like skin with a dark purple or yellow hue and subtle, minute white specks. Passion fruit is known for its unique flavor and nutritional properties, making it popular for fresh consumption and processing (Deshmukh et al. 2017). It is grown in various countries, including Sri Lanka, Kenya, Australia, Hawaii, India, the USA, New Guinea, South Africa, and Costa Rica. In India, it grows wild in parts of the Western Ghat, Himachal Pradesh, and northeastern states such as Meghalaya, Manipur, Nagaland, Mizoram, and Sikkim (*Deshmukh* et al. 2017).

Raw passion fruit contains 73% fluid, 22% carbohydrates, 2% protein, and 0.7% fat. Ripe passion fruit contains 97 cal per 100 g, 36% of the recommended daily intake (RDI) for vitamin C, 42% of the RDI for soluble fiber, 11% RDI for riboflavin, 10% RDI for niacin, 12% RDI for iron, and 10% RDI for phosphorus (Biswas et al. 2021). Citric and malic acids are the main causes of high acidity in passion fruit (pH 3.2) (Joseph‐Adekunle 2019). Additionally, the fruit is nutrient‐poor and rich in phytochemicals, such as carotenoids and polyphenols, as well as vitamins A, B2, and C. It is also high in proteins and minerals, including phosphorus (P), potassium (K), calcium (Ca), iron (Fe), sodium (Na), magnesium (Mg), sulfur (S), and chlorine (Cl) according to Deshmukh et al. (2017).

In addition to its antioxidant, anticonvulsant, antibacterial, anticancer, antidiabetic, antihypertensive, and anti‐sedative properties, *Passiflora edulis* also has numerous other favorable health impacts. Ansori et al. (2020) observed that in addition to its traditional applications as a sedative and sleeping pill, it is also a colon cleanser. Passion fruit contributes 52% to the residue within the juice business, and a whopping 85% of which is peel, with only 17% being seeds. Therapeutic passion fruit waste research reports. An example of a food supplement that exerts beneficial effects on blood glucose control is peel flour, which has been shown to enhance the health of the intestine by augmenting the synthesis of short‐chain fatty acids (Da Silva et al. 2014). Table 1 illustrates the results of in vivo studies, which revealed that passion fruit peel extract possesses antihypertensive activity (Lewis et al. 2013).

**TABLE 1:** Nutritional Composition of Purple Passion Fruit (*Passiflora edulis f. edulis*) Peel, Pulp, Juice, and Seeds.

Composition	Peel^a^ (per 1 g DW)	Pulp^b^ (per 1 g FW)	Juice (per 1 g FW)	Seeds (per 1 g DW)	References
Proximate					
Water (mg)	—	720–860	770–860	—	Ramaiya et al. (2019), U.S. Department of Agriculture (2019), Pruthi and Lal (1959)
Energy (kcal)	—	0.97	0.51	—	U.S. Department of Agriculture (2019), Pruthi and Lal (1959)
Protein (mg)	64.7–75.0	22.0–30.0	3.9–12.0	122–132	Dos Reis et al. (2018), Delvar et al. 2019, Ramaiya et al. 2019, Jiménez et al. (2011)
Total lipid (mg)	4.0–48.9	4.8–7.0	0.0–0.5	149–301	Dos Reis et al. (2018), Delvar et al. (2019), Ramaiya et al. (2018)
Carbohydrates (mg)	807	55.4–234	132–165	699	Dos Reis et al. (2018), Ramaiya et al. (2019),
Dietary fiber (mg)	617	39.2–104	1.0–2.0	438–551	Dos Reis et al. (2018), Ramaiya et al. (2018)
Ash (mg)	79.3	10.2–12.9	3.4–6.0	13.4–18.5	Dos Reis et al. (2018); Ramaiya et al. 2019
Minerals					
Calcium (mg)	3.10	0.12	0.04–0.18	0.06–1.73	Dos Reis et al. (2018), U.S. Department of Agriculture (2019)
Iron (mg)	0.046	0.016	0.0024–0.040	0.043–0.062	Dos Reis et al. (2018), U.S. Department of Agriculture (2019)
Magnesium (mg)	1.30	0.29	0.17	1.38–2.90	Dos Reis et al. (2018), U.S. Department of Agriculture (2019)
Phosphorus (mg)	0.70	0.68	0.63–1.15	—	Dos Reis et al. (2018), U.S. Department of Agriculture (2019)
Potassium (mg)	28.0	3.48	2.78	1.12–3.55	Dos Reis et al. (2018), U.S. Department of Agriculture (2019)
Sodium (mg)	0.073	0.28	0.06	0.048–2.41	Dos Reis et al. (2018), U.S. Department of Agriculture (2019)
Zinc (μg)	9.0	1.0	0.5	4.6–56	Dos Reis et al. (2018), U.S. Department of Agriculture (2019)
Vitamins					
Vitamin A (μg)	—	0.64	0.36	—	U.S. Department of Agriculture (2019)
Vitamin C (mg)	—	0.30	0.22–0.70	—	U.S. Department of Agriculture 2019
Vitamin E (μg)	—	0.2	0.1	—	U.S. Department of Agriculture (2019)

Abbreviations: −, data not available; DW, dry weight; FW, fresh weight.

^a^ Epicarp + mesocarp.

^b^ Without seeds.

The increasing importance of repurposing vegetable and fruit waste as long‐term providers of bioactive chemicals with potential nutraceutical uses has been explained in recent research (Jiménez‐Moreno et al. 2020; Panghal et al. 2021). Both mangosteen and passion fruits, as well as other underutilized species such as *Cnidoscolus aconitifolius* , contain bioactive compounds that address the increasing demand for functional foods (Anil et al. 2022; Chhikara and Panghal 2022).

**FIGURE 1:**
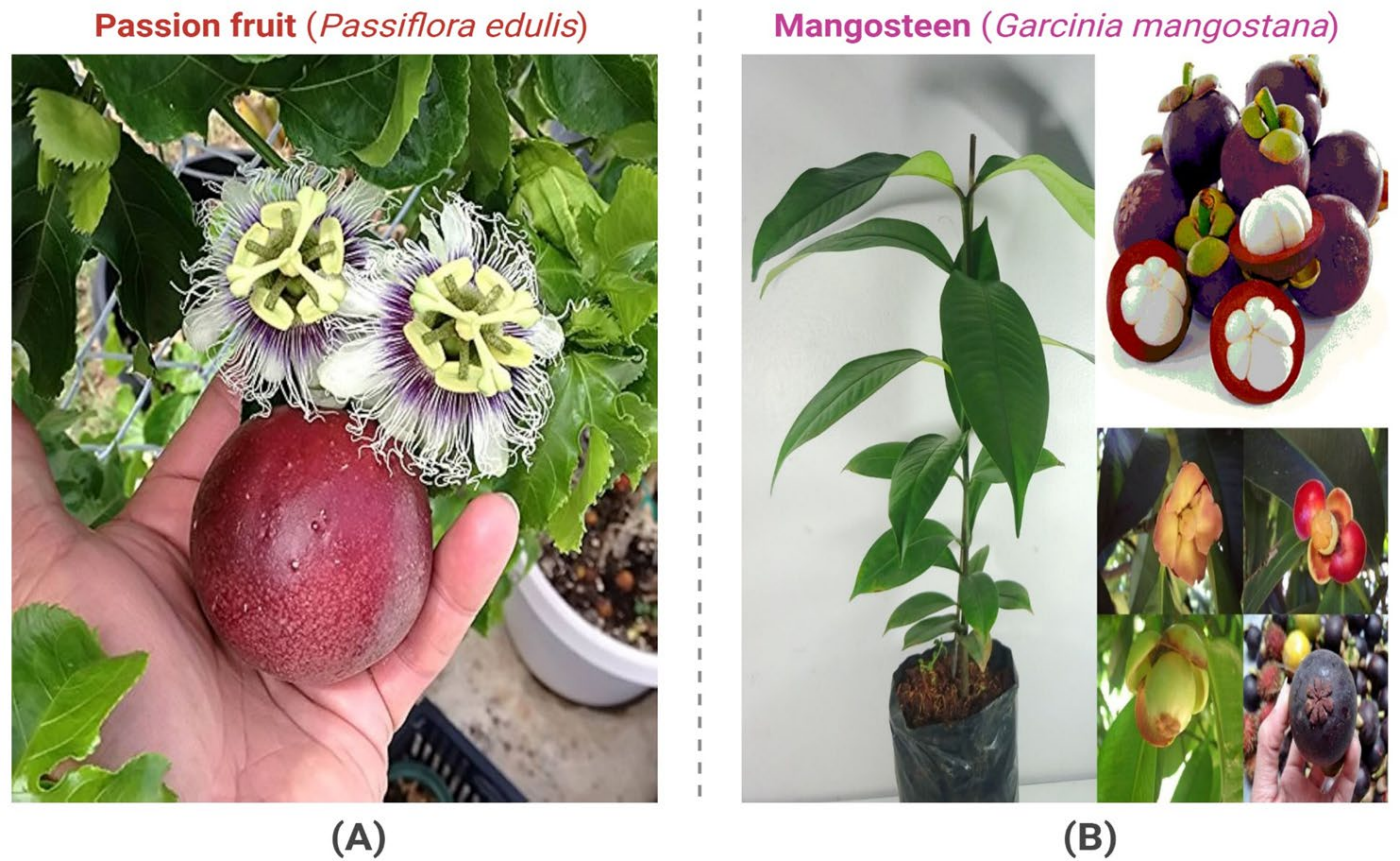
Visual Representation of Passion Fruit (A) and Mangosteen (B).

Mangosteen and passion fruit extracts may discover such potential based on the investigation of other plant bioactives, such as those of Acacia species, which have been successfully incorporated into the food and nutraceutical industries (Tiwari et al. 2023). In line with recent developments in other plant systems that are bioactive‐rich, such tropical fruits may become useful ingredients for medicines, cosmetics, and food because of their antioxidant, anti‐inflammatory, and metabolic health (Figure 1) (Jiménez‐Moreno et al. 2020; Panghal et al. 2021; Anil et al. 2022).

### 1.1 Objectives of the Review

The objective of this review was to comprehensively discover the pharmacological, industrial, and nutritional solicitations of passion fruit and mangosteen, the importance of their functional foods, and their therapeutic potential. This study investigated their pharmacological activities, including antioxidant, antidiabetic, anticancer, antibacterial, and anti‐inflammatory activities, by analyzing their dense phytochemical content, which consists of polyphenols, carotenoids, tannins, xanthones, and anthocyanins. The article also deliberates on their industrial importance in the food, beverage, and bakery industries, where their bioactive possessions and natural flavors improve food products. This review aims to highlight the broader uses of this tropical fruit in industry, nutrition, and health by integrating modern scientific investigations.

## 2 Pharmacological Perspectives of Mangosteen and Passion Fruits

### 2.1 Antioxidants Activity

Another vital aspect of mangosteen is its unique antioxidant profile, in which antioxidants counteract the adverse actions of free radicals linked to chronic diseases (Deshmukh et al. 2017). In addition to having high levels of antioxidant‐rich vitamins C and folate, mangosteen contains xanthones, a unique group of plant compounds endowed with powerful antioxidant activities (Sarian et al. 2017). Mangosteen extract showed total antioxidant activity in the Trolox equivalent antioxidant capacity (TEAC) test. The extract inhibited 50% of free radicals at 6.13 μg/mL (Ansori et al. 2021).

**FIGURE 2:**
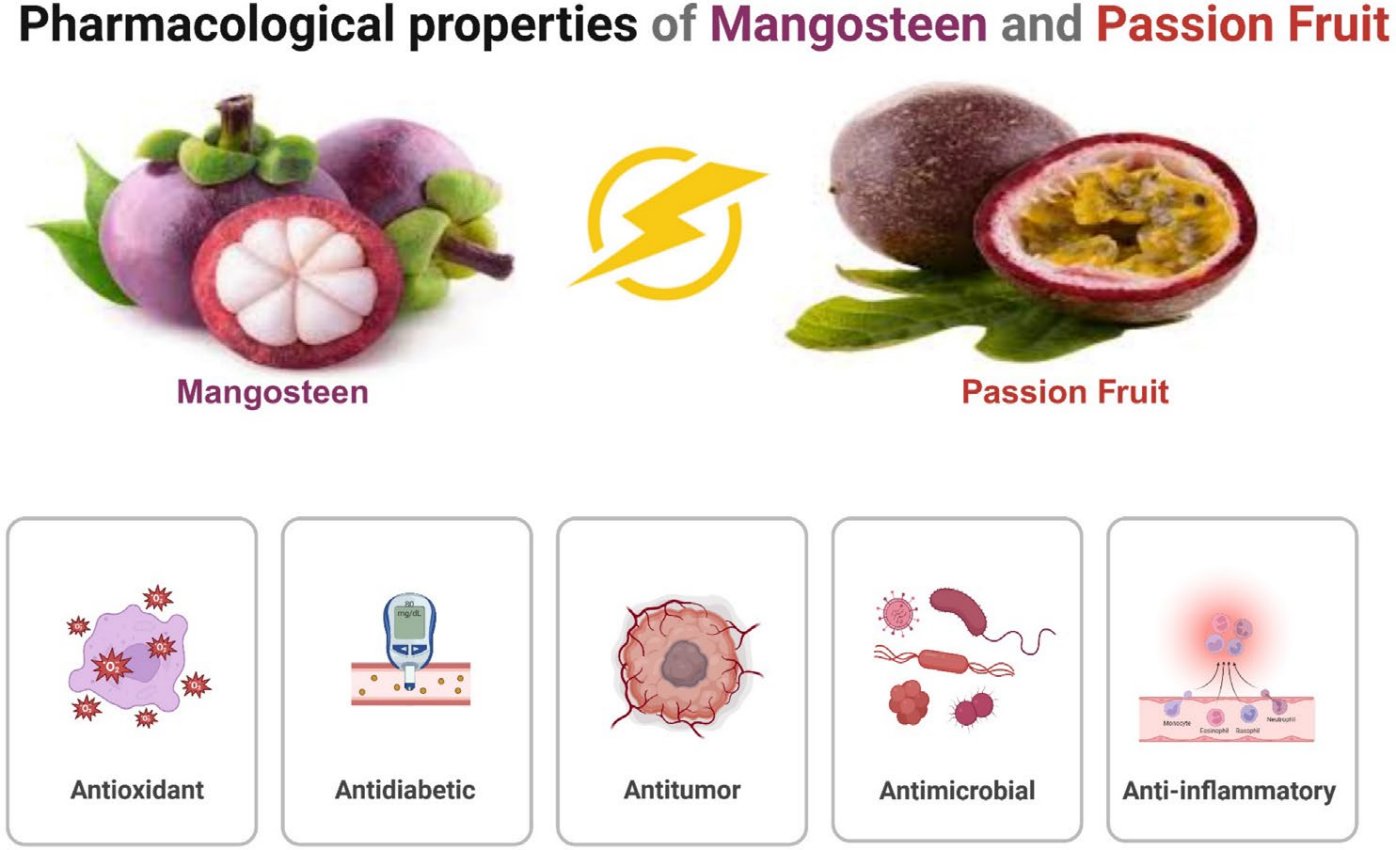
Pharmacological Properties of Mangosteen and Passion Fruit.

Husen, Kalqutny, et al. (2017), Husen, Khaleyla, et al. (2017), and Husen, Winarni, et al. (2017) also studied the antioxidant and antidiabetic activities of mangosteen pericarp extracts in streptozotocin‐induced diabetic rats. In addition, in the alpha‐mangostin test, Husen, Kalqutny, et al. (2017), Husen, Khaleyla, et al. (2017), and Husen, Winarni, et al. (2017) discovered that antioxidant activity is advantageous for improving the structure and function of the kidney in diabetic mice. Passion fruits are rich in antioxidants, which are crucial for the protection of the body from free radicals, enhance blood flow to the brain and nervous system, and reduce inflammation and cellular stress associated with diseases such as Alzheimer's and cardiovascular disease (Jusuf et al. 2020). In accordance with Panelli et al. (2018), male obese db/db mice treated with *P. edula* bark in vivo showed an increase in antioxidant capacity in plasma, kidney, liver, and adipose tissue, as well as reduced lipid oxidation in these tissues. In addition, Kandandapani et al. (2015) determined the antioxidant activity by increasing antioxidant enzymes in animal visceral organs following treatment with *P. edulis* leaves, seeds, and peel in streptozotocin‐induced diabetic rats (Figure 2).

### 2.2 Antidiabetic Activity

With 8.1 million Americans and 3.3 million Malaysians diagnosed with diabetes mellitus in 2015, cases of diabetes mellitus have increased at a high rate (Lima et al. 2016). The primary cause of such high blood glucose levels is impaired glucose metabolism, which is a long‐lasting disease characterized by reduced insulin secretion (Cheng et al. 2024; Control and Prevention 2014). Several studies have shown that mangosteen is antidiabetic and antihyperglycemic. Mangosteen pericarp extract effectively reduced lipid oxidation and high blood cholesterol in type 2 diabetic mice (Husen, Kalqutny, et al. 2017; Husen, Khaleyla, et al. 2017; Husen, Winarni, et al. 2017).

Ansori, Kuncoroningrat, et al. (2019) and Ansori, Susilo, et al. (2019) also found that in streptozotocin‐induced diabetic rats, chopping the mangosteen pericarp was renoprotective. Passion fruit is a good source of Ca, Mg, K, and Fe. Plant constituents such as polyphenols, which are also composed of piceatannol, are also present. Piceatannol increases insulin levels, which prevents diabetes, particularly type 2 diabetes in men (Chang et al. 2025). A number of studies, such as those conducted by Salles et al. (2020) and Soares and his coauthors, have shown that *P. edulis* peel flour, juice, and seeds can reduce blood glucose levels in diabetic rats and mice and thus cure diabetes (Salles et al. 2020; Soares et al. 2020). According to a study by Lima et al. (2016), ingestion of *P. edulis* peel flour increased glucose‐dependent insulinotropic polypeptide and glucagon‐like peptide‐1 levels. Increasing the rate at which glucose exited the diet also enhanced insulin sensitivity in obese rats fed a high‐fat diet. Finally, it prevented rats fed a low‐fructose diet from becoming insulin resistant (Lima et al. 2016).

### 2.3 Antitumor Activity

**FIGURE 3:**
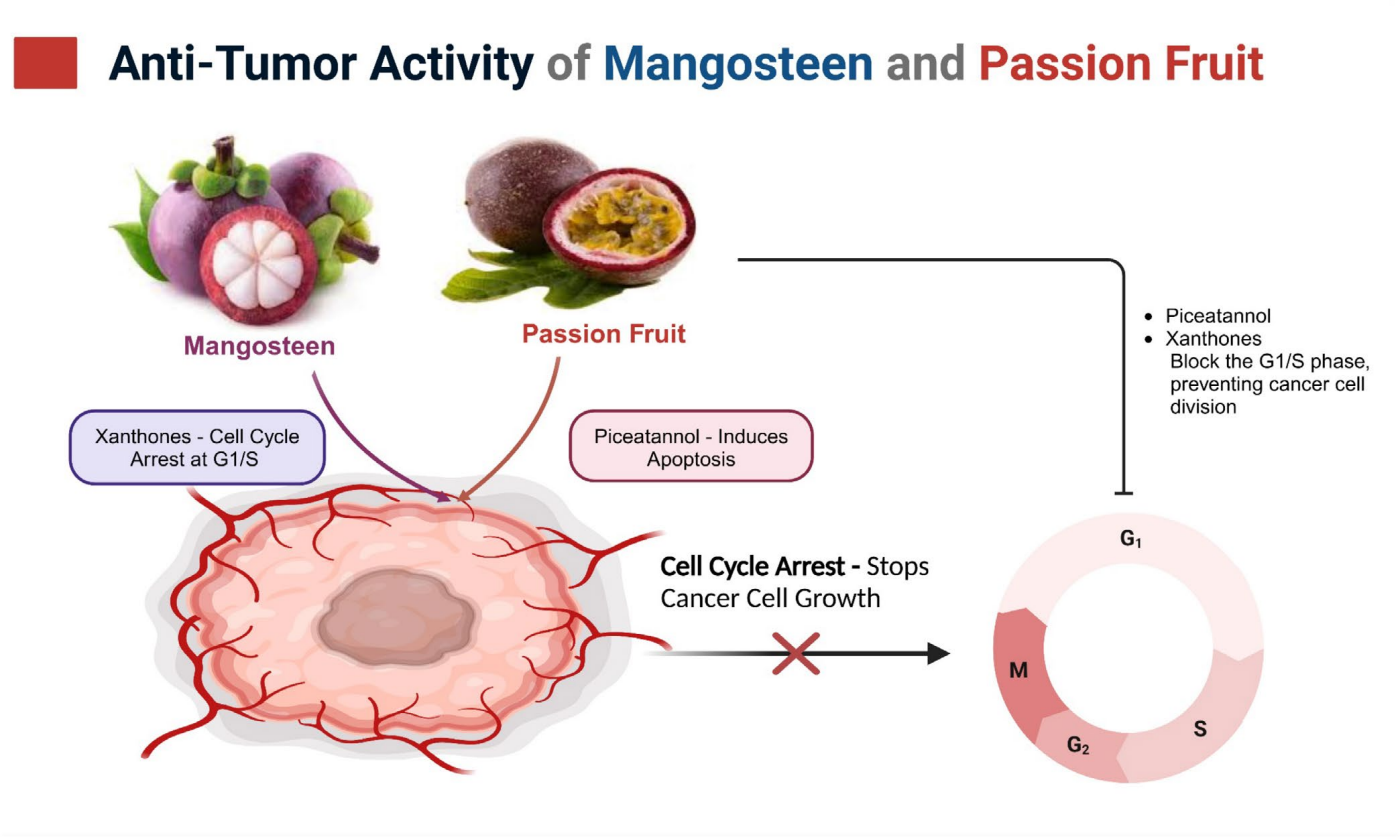
Antitumor Mechanism of Action of Passion Fruit and Mangosteen.

Population research has documented an association between a high diet of fruits and vegetables, including mangosteen, and fewer cancer cases. Phytochemicals in mangosteen, specifically xanthones, possess antioxidant and anti‐inflammatory activities, potentially making them naturally anticancer (Figure 3) (Gunter et al. 2020). Failla and Gutiérrez‐Orozco (2017) considered how α‐MG and other xanthones influence rodent xenograft growth and the capacity to kill cells and block cell proliferation in different cancer cell lines. Following subcutaneous injection of α‐MG (2 mg/kg), glioblastoma GBM8401 cells in BALB/c mice showed a 50% reduction in tumor growth, according to Chao et al. (2011). This action is accompanied by the induction of autophagy and enhancement of AMP‐activated protein kinase (AMPK) phosphorylation.

Nakagawa et al. (2007) showed that α‐mangostin can also cause caspase‐independent apoptosis by releasing endonuclease‐G from mitochondria, which results in cell death. It can also raise the expression of miR‐143 in DLD‐1 human colorectal cancer cells. Wang et al. (2011) also reported that α‐mangostin might be cytotoxic to the human melanoma SK‐MEL‐28 cell line. The movement of anticancer *P. edulis* has been the focus of most pharmacological studies. Piceatannol has potential anticancer properties against certain cancers, such as leukemia, breast cancer, and prostate cancer, and passion fruits have natural anti‐inflammatory and antioxidant capabilities (Wang et al. 2025). Owing to its high vitamin C and antioxidant content, passion fruit juice may help lower the risk of cardiovascular disease (Mota et al. 2018).

In male Balb/c mice implanted through Ehrlich carcinoma compartments, yellow passion fruit ethanol extract decreased tumor development by an inhibition frequency of 48.5% and improved mouse longevity to approximately 42%. The presence of intermediate and elongated chain fatty acids, including lauric acid, may be responsible for this (Mota et al. 2018). Sarcoma 180 tumor development was inhibited when the polysaccharide was administered orally or intraperitoneally at a reserve ratio of 40.59%–48.73% (Silva et al. 2012).

### 2.4 Antimicrobial Activity

Plants produce numerous secondary metabolites that protect themselves against pathogens, insects, and nematodes. Their antimicrobial properties have gained attention because of their resistance to antibiotics. Consumers also pressure the food industry to use natural compounds and extracts instead of synthetic preservatives. According to Suksamrarn et al. (2003), *Mycobacterium tuberculosis* is strongly inhibited by garcinone D, γ‐mangostin, mangostanin, α‐mangostin, and demethylcalabaxanthone. α‐Mangostin exhibits antibacterial efficacy against Methicillin‐resistant *Staphylococcus aureus* (MRSA) and vancomycin‐resistant Enterococci (VRE) (Sakagami et al. 2005).

As reported by Sudta et al. (2013), α‐MG analogs are more effective than the parent drug against *M. tuberculosis* . Moreover, the mechanism of action of xanthone has been adequately elucidated. The antibacterial and antifungal actions of passion fruit protect plants and individuals from bacterial and fungal infections. A peptide similar to 2S albumins from passion fruit seeds has antifungal properties against *Aspergillus fumigatus* , *Candida albicans* , *Trichoderma harzianum*, *Colletotrichum lindemuthianum*, *Fusarium oxysporum*, *Kluyveromyces marxiannus*, *Candida parapsilosis* , and *Saccharomyces cerevisiae* (*Jagessar* et al. 2017). In a thorough study, Ramaiya et al. (2014) revealed that antioxidant and phenolic constituents possess noteworthy antimicrobial properties, and the oil of yellow passion fruit seeds demonstrated antibacterial activity against *Salmonella enteritidis, Bacillus cereus, Staphylococcus aureus*, and *Escherichia coli* . The major components of the oil are tocopherol, n‐hexene, linoleic acid, and unsaturated fatty acids (Liang et al. 2023 *; Pereira* et al. 2019).

### 2.5 Anti‐Inflammatory Activity

The fruit shell of mangosteen has a modest impact on TNF‐α and IL‐4 production but is beneficial for treating inflammation‐related disorders by inhibiting NO and PGE2 emissions (Tewtrakul et al. 2009). Furthermore, α‐ and γ‐mangostins demonstrate anti‐inflammatory properties by preventing inducible NO synthase from functioning (Chen et al. 2008). It has been observed that α‐, β‐, and γ‐mangostin may decrease inflammatory responses in vitro in various professional and nonprofessional immune cell types (Table 2). In 2013, Gutierrez‐Orozco and Failla found that when human cell lines from different organs were exposed to a pro‐inflammatory stimulus, α‐MG (4–10 μM) abrogated the secretion of IL‐8 and TNF‐α. The anti‐inflammatory activity of 10 and 30 mg/kg of α‐ and γ‐MG was proven by Jang et al. (2012) to be equivalent in a mouse model of ovalbumin‐induced allergic asthma.

**TABLE 2:** Compounds of mangosteen and passion fruit and their mechanism of action.

Activity	Fruit source	Compounds	Mechanism of action	Benefits	Key findings	References
Antioxidant activity	Mangosteen	Xanthones, tannins, anthocyanins	Reduces oxidative stress and neutralizes free radicals	Protects against chronic diseases and aging	Significant improvement in antioxidant status in experimental models	Ansori et al. (2020), Failla and Gutiérrez‐Orozco (2017)
	Passion fruit	Polyphenols, carotenoids, flavonoids	Scavenges reactive oxygen species (ROS)	Prevents cellular damage and supports immune function	Enhances antioxidant enzyme activity	Han et al. (2025), Cazarin et al. (2015), López‐Vargas et al. (2013)
Antidiabetic activity	Mangosteen	α‐Mangostin, flavonoids	Modulates glucose metabolism, enhances insulin sensitivity	Reduces blood sugar levels	Demonstrated hypoglycemic effects in diabetic models	Husen, Kalqutny, et al. (2017), Husen, Khaleyla, et al. (2017), and Husen, Winarni, et al. (2017), Ibrahim et al. (2016)
	Passion Fruit	Pectin, dietary fiber, flavonoids	Regulates carbohydrate absorption, improves insulin response	Aids in glycemic control and reduces diabetes risk	Improved insulin sensitivity in diabetic rats	Lima et al. (2016), Kandandapani et al. (2015)
Antitumor activity	Mangosteen	α‐Mangostin, garcinone E	Induces apoptosis, inhibits cancer cell proliferation	Potential anticancer properties	Effective against colorectal and breast cancer cells	Nakagawa et al. (2007), Chao et al. (2011)
	Passion Fruit	Polyphenols, flavonoids	Suppresses tumor growth, induces cell cycle arrest	Reduces risk of various cancers	Cytotoxic activity against colon cancer cells	Arango Varela et al. (2017), Mota et al. (2018)
Antimicrobial activity	Mangosteen	Xanthones, tannins	Disrupts bacterial cell membranes, inhibits microbial enzymes	Effective against bacterial and fungal infections	Inhibitory effects on gram‐positive and gram‐negative bacteria	Jusuf et al. (2020), Jagessar et al. (2017)
	Passion Fruit	Flavonoids, alkaloids	Inhibits bacterial growth, disrupts microbial biofilm formation	Protects against infections	Antibacterial action against *Propionibacterium acnes*	Jusuf et al. (2020), Jagessar et al. (2017)
Anti‐inflammatory activity	Mangosteen	α‐Mangostin, γ‐Mangostin	Inhibits pro‐inflammatory cytokines (TNF‐α, IL‐6)	Reduces inflammation in chronic conditions	Suppresses inflammatory markers in various disease models	Chen et al. (2008), Jang et al. (2012)
	Passion Fruit	Flavonoids, alkaloids	Downregulates inflammatory pathways, reduces oxidative stress	Helps in treating inflammatory disorders	Decreased inflammation in colitis models	Cazarin et al. (2016), Herawaty and Surjanto (2017)

**FIGURE 4:**
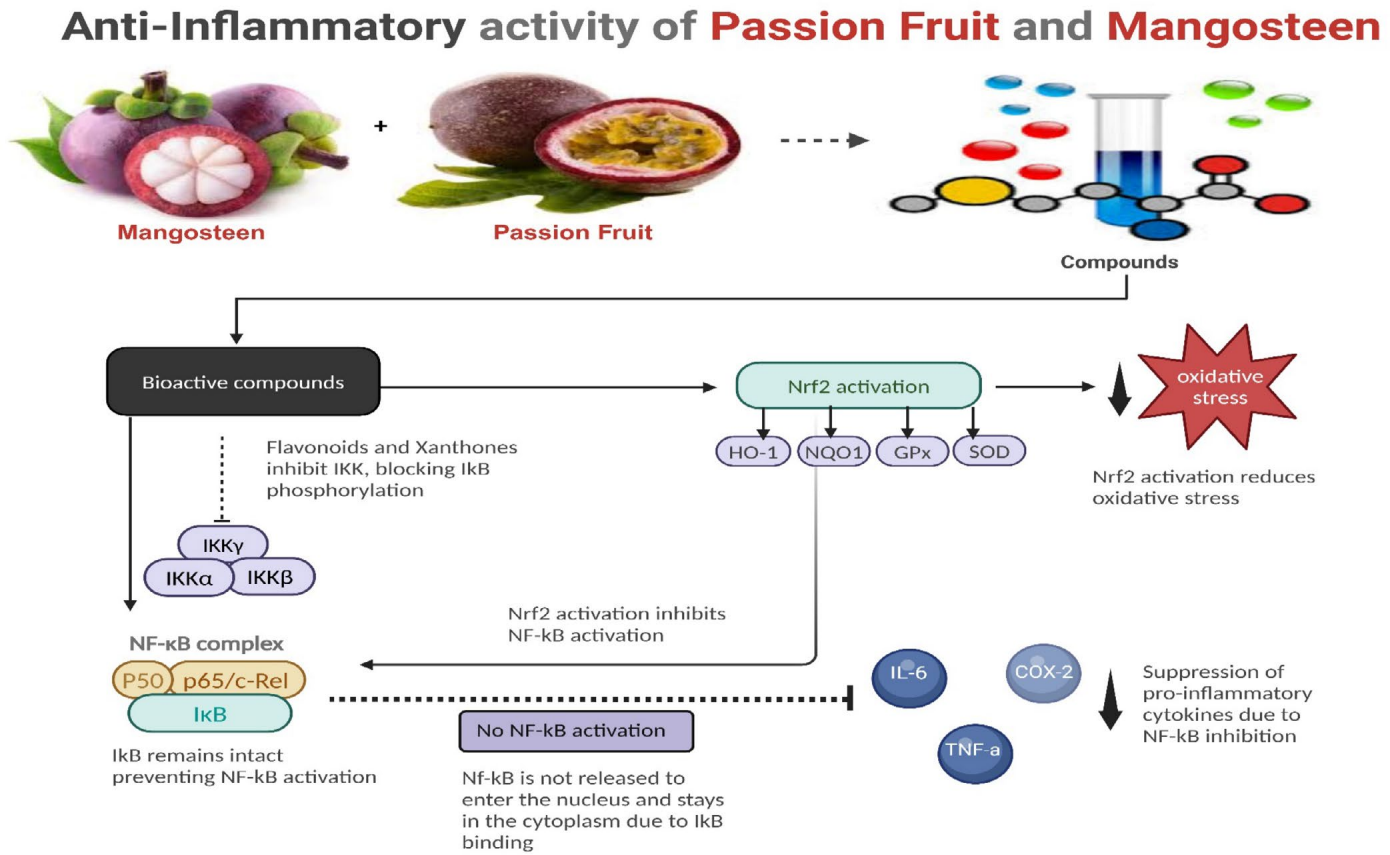
Anti‐Inflammatory Activity of Passion Fruit and Mangosteen.

Herawaty and Surjanto, in their 2017 study, examined the in vivo anti‐inflammatory activity of *P. edulis* extracts on inflammation induced by 2,4,6‐trinitrobenzene sulfonic acid, carrageenan, bradykinin, Substance‐P (SP), histamine, and dextran sodium sulfate (DSS). *Passiflora edulis* leaf aqueous extract lowered the pro‐inflammatory cytokines TNF‐a and IL‐1b in a rat model of colitis induced by 2,4,6‐trinitrobenzene sulfonic acid (Figure 4) (*Cazarin* et al. 2015). Figure 4 illustrates that in the dextran sodium sulfate‐induced mouse colitis model, *P. edulis* peel flour lowered the expression of the pro‐inflammatory cytokines TNF‐a, IL‐1b, IL‐6, IL‐12, and IL‐17.

## 3 Industrial Utilization of Mangosteen and Passion Fruit

Mangosteen ( *Garcinia mangostana* L.) is a popular fruit cultivated in many Asian nations. However, the growing popularity of this fruit has resulted in an abundance of abandoned pericarp. According to previous reports, 10 kg of harvested mangosteens produces 6 kg of pericarp (Anal 2017; Humaira et al. 2022). These pericarps are woody and contain bitter compounds with therapeutic benefits, including xanthones, tannins, and anthocyanins, which are used as nutritional supplements (Zhang 2018). The hypolipidemic, anti‐inflammatory, antimicrobial, and anti‐carcinogenic characteristics of these ingredients are all therapeutic advantages (Li, Liang, et al. 2025). The seeds of mangosteens, which contain 21.18% essential and non‐essential fatty acids, are another by‐product of the fruit preparation. They can be utilized as edible oils because it has been said that they are safe for the heart and liver (Anal 2017; Humaira et al. 2022).

Passion fruits are usually directly eaten and have an intense aroma. Passion fruits have a short shelf life and may deteriorate (Zhang 2018). Passion fruits are processed into various products owing to their health benefits and high phytonutrient content, including cake, jam, ice cream, yogurt, compound drinks, tea, soup stock, wine, vinegar, and condiment sauce (Li, Pan, et al. 2025; Zhang 2018). Passion fruit pulp, which is consumed or juiced, accounts for 51% of the fruit weight. Approximately 51% of the fruit weight is the pulp of the passion fruit, which is consumed or juiced. Thousands of tons of seeds, peels, and pulps are produced as agricultural co‐products owing to juice production. This waste is a burden to the environment. Peels are increasingly utilized in pectin production owing to economic development and environmental awareness, providing health benefits such as cholesterol reduction, hyperlipidemia, hypertension, glucose tolerance, and cancer prevention, and are also used in the pharmaceutical, cosmetic, and food industries (de Souza et al. 2018).

### 3.1 In Baking Industry

Because of their unique flavor profiles, bioactive properties, and prospective health benefits, passion fruit ( *Passiflora edulis* ) and mangosteen ( *Garcinia mangostana* L.) have expanded in popularity in the sphere of baking. A preferred fruit, mangosteen, is extremely desirable because of its sweet‐sour taste and fruitfulness for phytochemicals, including polyphenols, anthocyanins, and xanthones (Ansori et al. 2020). To improve the nutritional value and sensory suitability of baked foodstuffs, pericarp powder has been added to recipes (Palakawong and Delaquis 2018).

Mangosteen xanthones possess anti‐inflammatory and antioxidant properties that can possibly encompass the shelf life of lipids comprising baked foods by decreasing oxidative rancidity, as emphasized by Gunter et al. (2020). The scented pulp and high fiber, vitamin C, and flavonoid contents of the South American passion fruit also mark it as a preferred baked component (Wang et al. 2022 *; Biswas* et al. 2021). The seeds and rinds of the fruit, typically cast off as trash, can, according to López‐Vargas et al. (2013), be malformed into flours or extracts and exploited as components in baking desserts, jams, and pastry. Passion fruit peel extract was discovered to possess anxiolytic and antihypertensive properties, which are valuable in functional bakery foods that seek to enhance metabolic well‐being (Lewis et al. 2013).

Pectin and natural acidity in gluten‐free foods assist with moisture retention and texture in dough, which is an important advantage (Ramaiya et al. 2019). To combat problems such as spoilage and bitter aftertaste induced by mangosteen pericarps and render them more acceptable for application in commercial bakeries, further processing techniques such as microencapsulation or fermentation are required (Delvar et al. 2019). Future studies must focus on the suitability of bread products enriched with tropical fruits and the standardization of the extraction method.

### 3.2 In Beverage Industry

Xanthones, anthocyanins, and polyphenols comprise the humorous phytochemical composition of mangosteen, which is responsible for some of its antibacterial, anti‐inflammatory, and antioxidant activities (Ansori et al. 2020). The juice is sold as a functional fitness beverage that may prevent chronic diseases and enhance immunological activity (Gutierrez‐Orozco and Failla 2013). Mass commercialization is confusing because the fruit does not last long and has a limited shelf life (Palakawong and Delaquis 2018). To counter this, researchers are exploring the application of high‐pressure processing (HPP), freeze‐drying, and pasteurization to maintain the bioactive compounds in the product and increase its stability (Carias 2023).

Moreover, the claim for natural and organic drinks is being fulfilled by including mangosteen excerpts in flavored syrups, jams, and nutraceutical beverages. Passion fruits in elevation with acidity improve flavor and turn into a natural preservative; their powerful, tropical palate makes them a sought‐after component in most beverages (Biswas et al. 2021). Its juice is frequently blended with additional fruits, including mango and pineapple, to yield smoothies, alcoholic beverages, and other cold beverages (Zhu et al. 2017).

A valuable component in health beverages, the elevation of vitamin C and flavonoid content of the fruit also lends to its antioxidant and anti‐anxiety properties (Lewis et al. 2013). You can attain a great pact of flavor from passion fruit concentrate without breaking the bank; subsequently, even a small amount of energy goes a long way. Da Silva et al. (2014) reported that seed and peel leftovers revolve into fiber‐enriched constituents for probiotic drinks and herbal mixtures, which is consistent with maintainable trends (Table 3).

**TABLE 3:** Utilization of mangosteen and passion fruit and their benefits.

Sector	Fruit source	Utilized Part	Application	Benefits	References
Baking industry	Mangosteen	Pericarp extract	Natural food coloring	Rich in antioxidants	Ibrahim et al. (2016)
		Powdered peel	Added to bread and cakes	Enhances fiber content	Palakawong and Delaquis (2018)
		Pulp extract	Used in pastry fillings	Provides natural sweetness	Sumendap (2012)
		Freeze‐dried pulp	Incorporated into cookie dough	Increases nutritional value	Kusumaningrum et al. (2015)
	Passion Fruit	Seed flour	Used in gluten‐free baking	Improves texture and nutrition	Pereira et al. (2019)
		Pulp extract	Flavoring in muffins and pastries	Adds a tropical taste	López‐Vargas et al. (2013)
		Fiber powder	Added to bread and biscuits	Enhances dietary fiber	Lima et al. (2016)
		Passion fruit oil	Used in cake recipes	Provides essential fatty acids	Joseph‐Adekunle (2019)
Beverage industry	Mangosteen	Juice extract	Ingredient in health drinks	Rich in xanthones	Midin and Goh (2022)
		Powdered pericarp	Added to herbal teas	Enhances antioxidant properties	Panelli et al. (2018)
		Pulp concentrate	Used in fruit juice blends	Improves flavor and nutrition	Muhammad Ansori et al. (2021)
		Mangosteen rind infusion	Used in detox drinks	Anti‐inflammatory properties	Tewtrakul et al. (2009)
	Passion Fruit	Pulp juice	Base for fruit beverages	Rich in vitamins and minerals	Thokchom and Mandal (2017)
		Fermented pulp	Used in probiotic drinks	Supports gut health	Ramaiya et al. (2014)
		Passion fruit syrup	Used in cocktails and soft drinks	Natural flavor enhancer	Zhu et al. (2017)
		Passion fruit peel extract	Functional ingredient in energy drinks	Antioxidant‐rich	Soares et al. (2020)
		Passion fruit seed oil	Added to nutritional shakes	Source of omega‐6 fatty acids	Panelli et al. (2018)
		Passion fruit concentrate	Used in flavored iced teas	Enhances taste and aroma	Ma et al. 2024; Sakagami et al. (2005)
		Dehydrated passion fruit powder	Ingredient in instant beverages	Long shelf life	Kandandapani et al. (2015)
		Passion fruit pulp extract	Used in yogurt drinks	Adds tropical flavor	López‐Vargas et al. (2013)
		Passion fruit skin extract	Natural preservative in juices	Antimicrobial properties	Jusuf et al. (2020)

## 4 Conclusion and Future Perspectives

Two nutrient‐dense tropical fruits with high industrial and pharmaceutical value are mangosteen and passion fruits. Passion fruit's high content of vitamins and phytochemicals supports metabolic and cardiovascular well‐being, whereas the medicinal properties of mangosteen involve antibacterial and antioxidant effects owing to its bioactive compounds, such as polyphenols and xanthones. To meet the growing demand for natural health‐promoting factors, both fruits have industrial applications in functional foods, cosmetics, and flavorings. However, spoilage, limited cultivation, and waste by‐products (such as seeds and peels) remain. To enhance their social, economic, and health value, future research should explore new methods of extracting them, how to extend their shelf life, and how to use them in innovative ways.

The increased use of mangosteen and passion fruits as superfruits of functional value holds promise for further research and possible commercial applications. Agro‐practices that enhance productivity and climate resistance should be encouraged to maximize their potential, given the increased global demand. Maximization of the recovery of bioactive compounds from by‐products, such as peels and seeds, can be achieved using advanced extraction methods, including enzyme‐assisted and green solvents. This would minimize waste and simultaneously give nutraceuticals and cosmeceuticals added value.

The therapeutic potential of these fruits in the management of chronic conditions such as diabetes, cardiovascular disorders, and cancer needs to be established through clinical trials. If successful, such fruits may assume a central role in preventative healthcare. Their expanded use could also drive market growth in expanding food categories, such as functional drinks, plant‐based dairy alternatives, and sports nutrition. Demand could be further boosted if consumer education initiatives focused on flexibility and health benefits. Mangosteen and passion fruit could become a means of changing their historical applications to critical ingredients in the international functional food and drug industries. This may enhance human well‐being and sustainable economic growth by linking agricultural innovation, by‐product valorization, and evidence‐based health claims.

## Author Contributions

**Muhammad Tayyab Arshad:** writing – original draft (equal). **Nosiba S. Basher:** conceptualization (equal). **Nasir A. Ibrahim:** visualization (equal). **Ali Ikram:** methodology (equal). **Sammra Maqsood:** writing – review and editing (equal). **Amara Rasheed:** data curation (equal). **Feroza Naveed:** formal analysis (equal). **Muhammad Waqar:** visualization (equal). **Awais Raza:** conceptualization (equal). **Sana Noreen:** supervision (equal). **M. K. M. Al:** data curation (equal), resources (equal). **Muhammad Ahmad:** conceptualization (equal), data curation (equal). **Mahreen Faqeer Hussain:** project administration (equal). **Ali Jebreen:** validation (equal). **Ammar AL‐Farga:** data curation (equal), formal analysis (equal).

## Disclosure

The authors have nothing to report.

## Ethics Statement

The authors have nothing to report.

## Consent

The authors have nothing to report.

## Conflicts of Interest

The authors declare no conflicts of interest.

## Data Availability

The data that support the findings of this study are available from the corresponding author upon reasonable request.
